# Simulation of an Atypical Presentation of Necrotizing Enterocolitis in the Emergency Department

**DOI:** 10.7759/cureus.12604

**Published:** 2021-01-10

**Authors:** Jennifer Simpson, Maya I Brasher, Jennifer Arnold, Erin Endom, Cara B Doughty

**Affiliations:** 1 Pediatric Emergency Medicine, Baylor College of Medicine, Houston, USA; 2 Neonatal and Perinatal Medicine, University of Texas Health Science Center at Houston, Houston, USA; 3 Neonatal Medicine, Johns Hopkins All Children's Hospital, St. Petersburg, USA

**Keywords:** nec, pediatrics emergency, pediatric resuscitation, medical simulation, pals, neonatal sepsis, gi emergency, neonatal emergency

## Abstract

Necrotizing enterocolitis (NEC) is a gastrointestinal emergency most commonly seen in premature infants, but equally important to recognize in term infants. Early diagnosis and management is critical to achieving optimal patient outcomes. This report outlines a simulation of the challenging scenario of a term infant presenting to the emergency center with NEC as a result of bacteremia and sepsis due to a urinary tract infection (UTI). This simulation can be used for teaching different levels of learners including novice, intermediate, and advanced. It focuses on the presentation, diagnosis, and emergent management of NEC, and additionally incorporates Pediatric Advanced Life Support (PALS) for more advanced learners.

## Introduction

Necrotizing enterocolitis (NEC) is the most common acquired gastrointestinal emergency in the newborn infant, with significant morbidity and mortality. Approximately 90% of cases of NEC occur in preterm infants, with an incidence of about 12% in infants weighing less than 1500 grams at birth. About 10% of NEC cases occur in full-term babies (gestational age greater than 37 weeks) [1].

Classic presentation of NEC

The classic presentation often starts with a change in feeding tolerance and progresses to other abdominal symptoms and signs (including distention, tenderness, vomiting, hematochezia, and absent bowel sounds), along with non-specific systemic signs (including lethargy, apnea, temperature instability, and jaundice). Abdominal exam may reveal abdominal distension, discolored skin overlying the abdomen, or visible bowel loops.

Atypical presentation of NEC

Atypical presentations of NEC are more difficult to detect, especially when the signs and symptoms present subtly before fulminant illness develops. The age at onset of NEC is inversely related to gestational age, with the risk of NEC becoming highest around 3-4 weeks after birth in extremely preterm infants, compared to 5-6 days after birth in late preterm and term infants [2,3]. Compared to their preterm counterparts, term infants who develop NEC usually have a preexisting illness, including congenital heart disease (CHD), perinatal asphyxia, sepsis, respiratory failure requiring mechanical ventilation, or other high-risk conditions [1,4,5]. Additionally, these underlying conditions may further confound and delay the diagnosis of NEC in these infants.

Diagnosis and emergent management of NEC

Definitive diagnosis of NEC is made from plain abdominal radiographs showing either pneumatosis intestinalis, portal venous gas, or pneumoperitoneum. Neonates that are more mature (born at >28 weeks gestation) are more likely to show specific clinical and radiologic signs of NEC [3]. The modified Bell’s staging criteria summarizes the constellation of systemic, intestinal, and radiographic signs to diagnose NEC, and it provides an overview of treatment based on presentation and level of suspicion for suspected versus definite versus advanced NEC [6].

Emergent management for advanced NEC includes the establishment of intravenous access, initiation of empiric antibiotics including anaerobic coverage after obtaining blood culture, replogle tube placement for bowel decompression, and bowel rest. Fluid resuscitation is given as needed based on clinical condition, and inotropic and ventilatory support may also be required depending on severity [7,8]. Due to the rapidly progressive nature of NEC, a pediatric surgeon should be consulted for all cases at time of diagnosis; however, surgical intervention is reserved for patients with evidence of perforation, pneumoperitoneum, or clinical deterioration despite appropriate medical management [8,9].

Additional studies are done based on presentation but usually include a complete blood count, electrolytes, an initial point-of-care blood glucose, and blood gas with or without lactate. Laboratory findings associated with NEC include leukopenia or leukocytosis with left shift, thrombocytopenia, hyponatremia, hypoglycemia, and metabolic acidosis with elevated lactate [3,10]. Clinicians should have a low threshold to consider urinalysis and urine cultures, especially as bacteremia may be a contributing factor to the development of NEC [11]. If the infant is stable, cerebrospinal fluid studies should be considered. A cardiology consult should also be considered if the infant has a murmur, cardiomegaly on radiograph (XR), or history of CHD [12].

Morbidity and mortality of NEC

Early recognition and aggressive treatment of NEC results in improved clinical outcomes [13], but current statistics are still troubling. Overall mortality is 20%-30% (5%-20% among term neonates), with a higher mortality rate in infants with severe disease requiring surgical intervention [14]. Even among survivors, approximately half have long-term sequelae that range from gastrointestinal complications to impaired growth and poorer neurodevelopmental outcomes [15].

Importance of simulation

Thus, it is crucial for pediatric providers, especially providers outside of the neonatal intensive care setting, to experience this low-frequency, high-stakes event through simulation [16]. If a provider can recognize NEC early in its course and provide the appropriate management, he or she can make a substantial difference in that child’s clinical outcome.

The purpose of this simulation is to educate learners about NEC when it presents atypically, particularly in settings such as the emergency center where providers may not be accustomed to including this disease in their differential diagnosis. The case also teaches learners to anticipate, detect, and respond to a patient’s clinical deterioration.

## Technical report

Objectives for novice learners

1. Perform a focused history and physical exam.

2. Develop a differential diagnosis, request appropriate diagnostic studies, and interpret imaging and laboratory tests to diagnose NEC. Be able to determine the stage of NEC based on the modified Bell's criteria to determine appropriate management (Table 1).

**Table 1:** Modified Bell's Staging Criteria for the Diagnosis and Management of NEC in Term and Preterm Neonates [6] Nothing by mouth (NPO); necrotizing enterocolitis (NEC).

Stage	Systemic Signs	Intestinal Signs	Radiologic Signs	Treatment
Suspected IA	Temperature instability, apnea, bradycardia	Increased residuals, mild abdominal distention, occult blood in stool	Normal or mild ileus	NPO, antibiotics x3 days
Suspected IB	Same as IA	Same as IA, plus gross blood in stool	Same as IA	Same as IA
Definite IIA, Mildly ill	Same as IA	Same as I, plus absent bowel sounds, abdominal tenderness	Ileus, pneumatosis intestinalis	NPO, antibiotics x7-10 days
Definite IIB, Moderately ill	Same as IA, plus mild metabolic acidosis, mild thrombocytopenia	Same as I, plus absent bowel sounds, definite abdominal tenderness, abdominal cellulitis, right lower quadrant mass	Same as IIA, plus portal vein gas, with or without ascites	NPO, antibiotics x14 days
Advanced IIIA, Severely ill, Bowel intact	Same as IIB, plus hypotension, bradycardia, respiratory and/or metabolic acidosis, disseminated intravascular coagulation, neutropenia	Same as I and II, plus signs of generalized peritonitis, marked abdominal tenderness and distension	Same as IIB, plus definite ascites	NPO, antibiotics x14 days, fluid resuscitation, inotropic support, ventilator therapy, paracentesis
Advanced IIIB, Severely ill, Bowel perforated	Same as IIIA	Same as IIIA	Same as IIB, plus pneumoperitoneum	Same as IIA, plus surgery

3. Perform emergent management of NEC, including selection of intravenous (IV) antibiotics and calling for a surgical consultation.

4. Utilize effective team communication to distribute the workload and express a shared mental model throughout the development of a differential diagnosis, narrowing the differential, initial management, and subsequent clinical deterioration.

Objectives for intermediate learners

1. All objectives above, and:

2. Demonstrate skills appropriate for initial management of NEC including placement of replogle tube.

3. Anticipate and identify an acute deterioration in the infant’s condition.

4. Set priorities dynamically to respond to changes in clinical status.

5. Provide appropriate resuscitative treatment in response to deterioration, such as obtaining intraosseous (IO) access to administer fluid resuscitation.

Objectives for advanced learners

1. All objectives above, and:

2. Accurately interpret diagnostic studies.

3. Provide advanced airway management, including bag-valve-mask ventilation and intubation.

4. Provide advanced cardiovascular support such as blood pressure support.

This scenario takes place in a simulated emergency room of a community hospital or a pediatric tertiary care center, depending on the instructor's preference and the desire to have specialists available on site. The room setting is a resuscitation bay with access to full monitors, airway equipment, and resuscitation cart. For intermediate and advanced learners, replogle and IO kit may also be available. The patient is a newborn or infant mannequin, and there is also one standardized patient (also called simulated parent, or SP) acting as the parent historian. Supporting laboratory and radiologic findings should be provided to facilitate the simulation.

Pre-briefing

Trainees should receive an introduction to the mannequin, including a review of any technical issues or limitations with the mannequin, and limitations with resource availability and consultants available based on your chosen location. Expectations should be based on the learner's level of training. In addition, the pre-briefing should includes elements that help establish psychological safety and include reminders about learner confidentiality and the basic assumption being held for all participants, namely that all participants are intelligent, capable, care about doing their best and want to improve [17]. The pre-briefing should also include reminders to immerse fully in the scenario and uphold the fiction contract, which is the agreement between participants and instructors to proceed as if the simulation is real while simultaneously acknowledging that it is not real [18]. Subsequently, a pre-briefing of the case specifics should be given and may include the following dialogue:

“Your group is an interdisciplinary team working in a large pediatric emergency center. The team leader is the attending who just signed up to see a new patient. The triage nurse has reported that the newborn infant was initially brought in for jaundice, but he was just moved to a resuscitation room after the nurse noted a rectal temperature of 100.5°F on the initial vital signs. The baby's caregiver is at the bedside. Your job is to assess the infant and provide any necessary interventions. For this scenario, you should verbalize any laboratory or imaging studies that your team desires, and results will be given to you when they are available.”

Case

The learners in this scenario will be facing the challenging task of diagnosing NEC in a previously healthy term infant. The first half of the scenario will require a brief but important history and physical exam, after which the team should develop a differential diagnosis for the ill-appearing neonate, followed by ordering appropriate studies and performing appropriate initial stabilization measures. Once the diagnosis of NEC is made from the abdominal radiograph, the team should perform the initial management of NEC while calling for a surgical consultant. Based on the team size, available equipment, and roles of individuals involved (i.e. physicians, nurses, etc.), the learners may either verbalize or physically complete their treatment steps.

Either after the surgical consult is called or after five minutes, the scenario will transition to the second half, which will require the learners to implement their training in Pediatric Advanced Life Support (PALS). They will need to first address respiratory failure, which will require assisted ventilation that progresses to intubation. They will also need to address decompensated septic shock requiring fluid resuscitation. The case will conclude once the patient has been stabilized.

Patient’s background data and baseline state

History of present illness: Patient is a 4-day-old term male who presents with one day of increased sleepiness and poor feeding. Mother has struggled with breastfeeding and gave formula on day of life 2 while waiting for her milk supply to increase. She has been trying to exclusively breastfeed since day of life 3. However, the infant has not been waking up to feed for the last 12 hours. The parents attempted to feed him formula eight hours ago, but he spit up the entire feed. He has had two wet diapers and two stools over the last 24 hours.

Medical history: Born at 38 weeks by spontaneous vaginal delivery to a 22-year-old primigravid mother. Family does not recall appearance, pulse, grimace, activity, respiration (APGAR) scores. Mother received routine prenatal care, took no medications except for prenatal vitamins, and denies drugs and alcohol. No complications during the pregnancy or delivery. Infant was discharged home at the same time as the mother. No previous surgeries.

Review of systems: Sleeping more, fussy, decreased appetite, spitting up of feeds, decreased urine output, jaundice

Current medications and allergies: None

Physical Examination

General: Lethargic.

Weight, Height: 2.9 kg, length unknown

Vital signs (VS): heart rate (HR) 170 beats per minute (bpm); blood pressure (BP) 80/45 mmHg; respiratory rate (RR) 60 breaths per minute; oxygen saturation (SpO2) 98% on room air; temperature (T) 100.5°F (38.1°C)

Head: Anterior fontanelle is sunken. No facial anomaly.

Eyes: Normal conjunctivae. No scleral icterus.

ENT: Dry mucous membranes. Oropharynx is clear.

Neck: Supple.

Lungs: Effort normal and breath sounds normal. No respiratory distress.

Heart: Tachycardia. Regular rhythm. Palpable pulses. Capillary refill 3 seconds.

Abdomen: Full and diffusely tender to palpation. Unable to palpate liver or spleen.

Genitourinary: Uncircumcised.

Musculoskeletal: No edema or deformity.

Skin: Warm and dry. Jaundiced to abdomen. No cyanosis, mottling, or pallor. Not diaphoretic. No petechiae, pallor, ecchymosis, or rash.

Baseline Simulator State

Vitals: HR 170 bpm; BP 80/45 mmHg; RR 60; SpO2 98% on room air; T 100.5°F (38.1°C)

Respiratory: No baseline alterations.

Cardiovascular: Tachycardia, normal sinus rhythm.

Gastrointestinal: Distended (if mannequin allows; otherwise should be given verbally during physical exam)

Skin: Jaundiced.

Neurologic: Difficult to arouse.

Simulation

**Figure 1:**
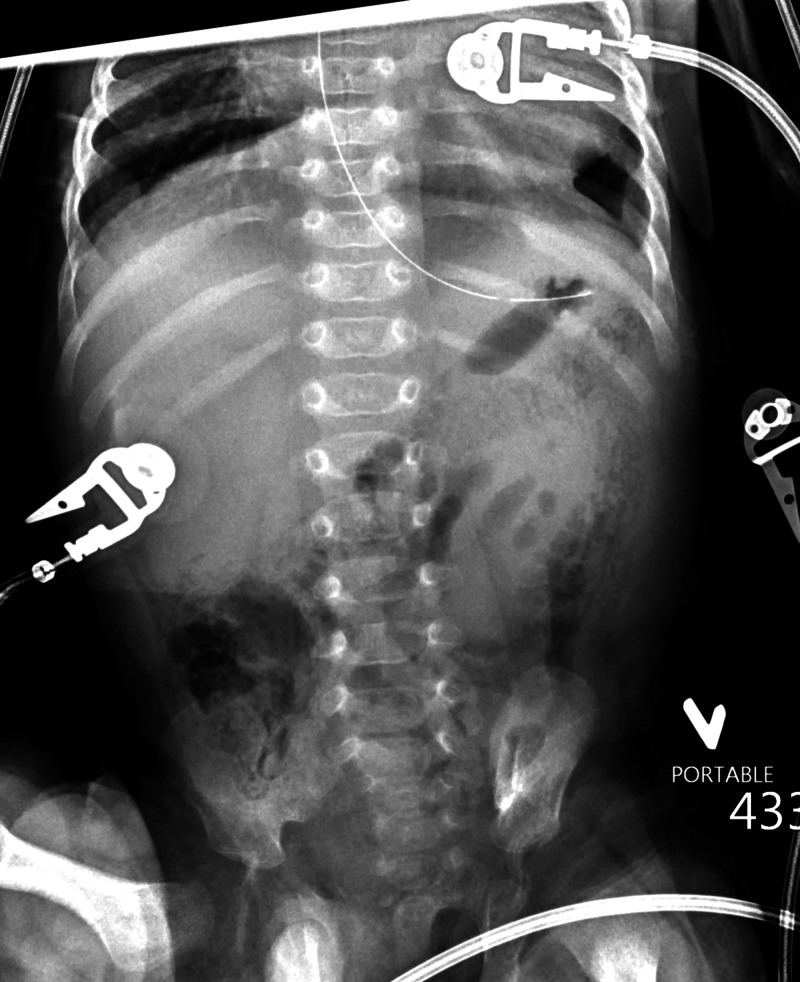
Anteroposterior Abdominal XR Showing Diffuse Pneumatosis Intestinalis (Courtesy of Scott Dorfman, MD)

**Figure 2:**
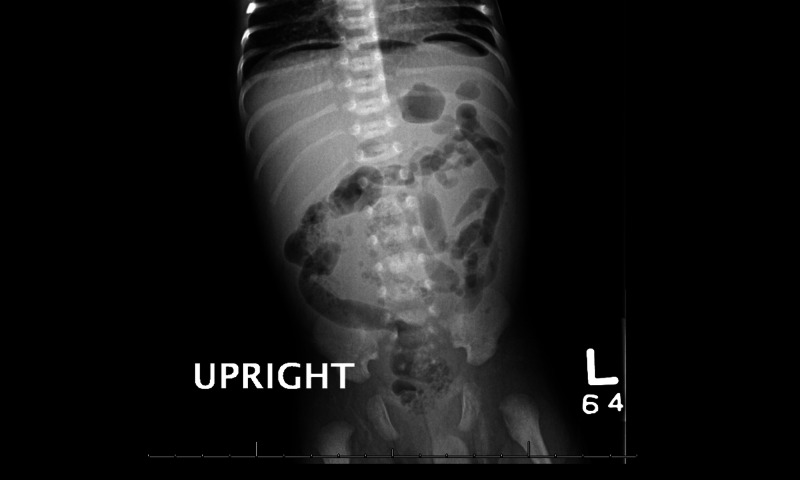
Upright Abdominal XR Showing Pneumoperitoneum

Below is a stepwise, detailed scenario of the learner actions, mannequin operator, and SP responses to execute the simulation (Table 2). Use supporting laboratory data, as seen in Table 3, and radiographs to facilitate the simulation (Figures 1-2).

**Table 2:** Stepwise, Detailed Scenario of Learner Actions, Mannequin Operator, and Simulated Parent Responses Airway-breathing-circulation (ABC), point-of-care (POC), lumbar puncture (LP), bag-valve-mask ventilation (BVM), necrotizing enterocolitis (NEC), simulated parent (SP), vital signs (VS), heart rate (HR), blood pressure (BP), respiratory rate (RR), oxygen saturation (SpO2), temperature (T), nothing by mouth (NPO), Pediatric Advanced Life Support (PALS), intravenous (IV).

Clinical State	Patient/Mannequin Status	Learner Actions	Operator Responses
1. BASELINE	Patient is either in parent’s arms or in crib. Sunken fontanelle, dry mucous membranes, jaundiced, distended and tender abdomen, capillary refill 3 seconds. Has weak cry when abdomen is palpated. Optionally, IV access may be in place. VS: HR 170 bpm; BP 80/45 mmHg; RR 60; SpO_2_ 98% room air; T 100.5°F (38.1°C)	1. Move baby to bed if needed and obtain initial vital signs. 2. Review ABC to ensure that patient is stable. 3. Obtain brief history and perform physical exam. 4. Verbalize differential diagnosis including necrotizing enterocolitis. All team members can participate, but the leader is responsible for mental modeling. 5. Verbally request POC glucose, as well as blood and urine tests including cultures. 6. Request portable abdominal XR. 7. Interpret XR to confirm diagnosis; optionally could be called with diagnosis by radiologist, especially to assist novice learners. 8. Call for surgical consultation. 9. Verbalize that patient is NPO. 10. Request or place replogle tube. 11. Initiate broad spectrum antibiotics to cover for aerobic and anaerobic bacteria. 12. Request peripheral IV and bolus of normal saline (20mL/kg or 60mL here). 13. Recognize that LP should be deferred given risk of deterioration.	1. Maintain patient in baseline state. 2. If wireless mannequin is available, baby should start the simulation in the parent’s lap and not be moved to the crib unless requested. 3. SP in the caregiver role will give history as requested by learners. Should emphasize that the infant has not fed for 12 hours and vomited after attempted feed 8 hours ago. SP can also report to the team as needed the physical exam aspects that cannot be readily detected on the mannequin (i.e. dry mouth, infant is sleepy). 4. If the learner painfully stimulates the baby or palpates the abdomen, the operator should activate the “weak cry” feature of the mannequin. 5. If learner requests a POC glucose, results should be given immediately (71). 6. If learner requests other laboratory tests, results should be reported as “in progress”. 7. If learner requests imaging, provide visual or verbal results after appropriate time frame. May use Figure 1 or Figure 2 below. 8. When team requests antibiotics, direct them to specify which antibiotics. 9. Surgical consultation can be an informal phone call or a full consultation. 10. Transition to Unstable Condition after five minutes OR after surgical consult called.
2. UNSTABLE – respiratory and circulatory decompensation	Patient becomes hypoxic and hypotensive with delayed capillary refill (5 seconds) and mottling. VS: HR 190 bpm; BP 55/25 mmHg; RR 4; SpO_2_ 75% on room air; T unchanged (100.5°F/38.1°C)	1. Identify and verbalize acute onset of bradypnea/hypoxia. 2. If not done previously, the team leader should assign roles as the situation has become emergent. 3. Revisit ABC’s – stop at breathing when abnormality detected. 4. Open the airway and monitor for improvement. 5. Set up BVM and begin ventilating adequately. 6. Troubleshoot BVM if not done appropriately. 7. Recognize BVM is not sufficient and prepare to intubate. 8. Perform intubation. 9. Confirm endotracheal tube placement. 10. Team should continue good communication and mental modeling of his diagnosis of respiratory failure.	1. Start by decreasing RR to 4 with shallow respirations. Then, make infant hypoxic with SpO_2_ 80s and recover after a few seconds. If team does not notice the desaturation, can repeat once. Finally make persistently hypoxic with tachycardia and hypotension. If learner attempts painful stimulation or abdominal palpation, there should be no response. 2. If ventilation with BVM is not adequate, then SpO_2_ should continue to decline. 3. If ventilation with BVM is appropriate, then SpO_2_ should increase, but not higher than 89. 4.If team uses BVM for five minutes without intubation (and no replogle tube has been placed), SpO_2_ should rapidly decline. 5. If team intubates into the esophagus (no replogle tube placed), SpO_2_ should rapidly decline. 6. If diagnosis of underlying NEC has not yet occurred, it can be diagnosed if a post-intubation chest XR is requested. The team should simply be called by the “radiologist” who tells them the XR findings (Figure 1).
3. UNSTABLE – circulatory decompensation	Respiratory symptoms stabilize with intubation, but patient remains hypotensive with delayed capillary refill (5 seconds). VS: HR, 190 bpm; BP 55/35 mmHg, RR – rate of bagging; SpO_2_ 100% with intubation; T unchanged (100.5°F/38.1°C)	1. Continue ABC’s - a second learner should request a new blood pressure or check pulses and capillary refill. 2. Verbalize diagnosis of decompensated septic shock. If not previously obtained, IV access should be requested, and a normal saline fluid bolus given (20 mL/kg or 60mL here). 3. If diagnosis of NEC was discovered on post-intubation film, learners should perform initial management steps: place replogle tube, consult general surgery, verbalize or draw cultures, and verbalize or give broad spectrum antibiotics including anaerobic coverage.	Note that this may happen after state #2 or simultaneously with state #2 depending on size of team. Team should utilize PALS algorithm – proper management of decompensated septic shock [19]. After 2^nd^ normal saline bolus given, progress to state #4 (“stabilized”).
4. STABILIZED	VS: HR, 150 bpm; BP 75/45 mmHg, RR – rate of bagging; SpO_2_ 100% with intubation; T unchanged (100.5°F/38.1°C)	Patient is now stable after intubation and IV fluid resuscitation. Scenario ends.	Patient is now stable after intubation and IV fluid resuscitation. Scenario ends.

**Table 3:** Laboratory Studies to be Printed or Displayed on Screen in Simulation Lab and Provided to Trainees When Requested Partial pressure of carbon dioxide (pCO2), partial pressure of oxygen (pO2), white blood cells (WBC).

Laboratory Test	Results	Normal Range
Total Bilirubin (mg/dL)	14	0.2 - 1.3
Glucose (mg/dL)	71	60 - 90
Urine Analysis (Dipstick)		
Color	Orange	Yellow
Appearance	Turbid	Clear
Glucose	Trace	Negative
Bilirubin	2	Negative
Ketones	Negative	Negative
Specific Gravity	1.025	<= 1.030
Blood	Small	Negative
pH	6.0	5.0 - 8.0
Protein	1+	Negative
Nitrite	Positive	Negative
Leukocyte Esterase	Positive	Negative
Venous Blood Gas		
pH	7.24	7.28 - 7.42
pCO2 (mmHg)	60	38 - 52
pO2 (mmHg)	52	20 - 49
Bicarbonate (mmol/L)	22	22 - 26
Base Excess (mmol/L)	-8	-2 - 2
Metabolic Panel		
Sodium (mEq/L)	133	135 - 145
Potassium (mEql/L)	4.5	3.5 - 5.1
Glucose (mg/dL)	71	41 - 90
Ionized Calcium (mmol/L)	1.25	1.05 - 1.25
Lactate (mmol/L)	4.5	0.5 - 2.2
Complete Blood Count and Differential		
WBCs (10^3^/microL)	3.08	9.1 - 34
Hemoglobin (g/dL)	17.5	15.0 - 19.6
Hematocrit (%)	53.0	45.0 - 58.8
Platelets (10^3^/microL)	98	150 - 450
Segmented neutrophils (%)	30.0	32.0 - 67.0
Lymphocytes (%)	40.0	25.0 - 37.0
Bands (%)	8.0	0 - 8

## Discussion

This scenario presents learners with multiple challenges both in terms of diagnosing NEC with an atypical presentation as well as the more generalizable challenge of responding to a clinical deterioration. The debriefing session should discuss these challenges sequentially as they follow the natural course of the scenario.

Diagnosis and emergent management of NEC

In the first part of the scenario, the learners must integrate cues from the history (feeding intolerance, increased sleepiness) with the physical exam (abnormal vital signs, signs of dehydration, abnormal abdominal exam, jaundice) and lab findings (leukopenia, thrombocytopenia), and finally order the key diagnostic test (abdominal XR) to reach the diagnosis of NEC. This multi-step process is not straightforward as the learner may be distracted by certain findings, such as the infant’s jaundice or fever, and pursue other diagnostic or treatment pathways, such as empiric evaluation and treatment for neonatal fever, or treatment for urinary tract infection (UTI) without realizing the associated complication. An ill-appearing neonate has a broad differential, which includes infectious etiologies (sepsis, UTI, meningitis), congenital heart disease, intra-abdominal pathologies, endocrine disorders (such as congenital adrenal hyperplasia), electrolyte abnormalities (such as from formula mixing errors), inborn errors of metabolism, and trauma including non-accidental trauma.

Specific intra-abdominal pathologies can be divided into obstructive and infectious etiologies. Obstructive etiologies which should be considered include volvulus, Hirschsprung's disease, meconium ileus, or other congenital bowel atresia or obstruction. Infectious etiologies to consider include NEC or a septic ileus [20]. Spontaneous intestinal perforation may also result in an acute abdomen but occurs in preterm infants. Discussion of this differential diagnosis should also include the subtle variations in presentation and work-up. In general, initial imaging for an infant presenting to the ED with abdominal distension or feeding intolerance would be a plain abdominal radiograph, before the differential diagnosis is further refined.

The debriefing should include a discussion of mental modeling that either did or did not lead to a correct diagnosis, and participants can reflect on the role of distraction or bias in the development of their differential diagnosis and diagnostic steps. The steps for emergent management of advanced NEC can also be reviewed: establishment of venous access, initiation of empiric antibiotics including anaerobic coverage, replogle tube placement, bowel rest, and consideration of fluid resuscitation, inotropic and ventilatory support. Simultaneously, treatment must include a consultation to pediatric surgery, or in the case of a community hospital, transportation to a facility with pediatric surgery.

Responding to a clinical change

In the second half of the scenario, the learner must anticipate and respond to the patient’s clinical deterioration. This more generalizable challenge integrates the cognitive and technical skills of the response, such as PALS, in addition to setting appropriate priorities and utilizing effective team communication. The debriefing should focus on the transition from the initial management to the resuscitative efforts, with emphasis on detection of the clinical change and any delays that may have occurred. Was clinical worsening anticipated based on their understanding of the patient’s diagnosis? Was there any delay due to a failure to anticipate possible clinical decline? How did the team’s communication/mental modeling of this change affect their subsequent response? If a correct diagnosis was not reached in the initial part of scenario, participants can discuss their response when the diagnosis was revealed. The specific knowledge goals of this simulation regarding the diagnosis and management of NEC can then be discussed in this second section, and learners can be evaluated after the debriefing to determine knowledge retention.

## Conclusions

Prompt recognition of NEC is essential as it has potential for both high morbidity and mortality. This scenario integrates important skills that teach both specific medical knowledge regarding NEC as well as more generalizable skills. Various learners can benefit from the scenario with the suggested adaptations. Through this scenario, providers will gain experience with the low-frequency, high-stakes event of the atypical presentation of NEC outside of the neonatal intensive care unit.
